# Bone Histomorphometry and ^18^F-Sodium Fluoride Positron Emission Tomography Imaging: Comparison Between only Bone Turnover-based and Unified TMV-based Classification of Renal Osteodystrophy

**DOI:** 10.1007/s00223-021-00874-9

**Published:** 2021-06-17

**Authors:** Louise Aaltonen, Niina Koivuviita, Marko Seppänen, Inari S. Burton, Heikki Kröger, Eliisa Löyttyniemi, Kaj Metsärinne

**Affiliations:** 1 Department of Medicine, Kidney Center, Turku University Hospital, PL 52, Kiinamyllynkatu 4-8, 20521 Turku, Finland; 2 Turku PET Centre, University of Turku, Kiinamyllynkatu 4-8, 20521 Turku, Finland; 3 Department of Clinical Physiology, Nuclear Medicine, Turku University Hospital, PL 52, Kiinamyllynkatu 4-8, 20521 Turku, Finland; 4 Kuopio Musculoskeletal Research Unit (KMRU), Institute of Clinical Medicine, University of Eastern Finland, POB 1627, Kuopio, Finland; 5 Kuopio University Hospital, Kuopio, Finland; 6 Department of Biostatistics, University of Turku, Kiinamyllynkatu 10, 20014 Turku, Finland

**Keywords:** Bone histomorphometry, ^18^F-NaF-PET, Hyperparathyroidism, Renal osteodystrophy, Bone turnover, CKD

## Abstract

Bone biopsy is the gold standard for characterization of renal osteodystrophy (ROD). However, the classification of the subtypes of ROD based on histomorphometric parameters is not unambiguous and the range of normal values for turnover differ in different publications. ^18^F-Sodium Fluoride positron emission tomography (^18^F-NaF PET) is a dynamic imaging technique that measures turnover. ^18^F-NaF PET has previously been shown to correlate with histomorphometric parameters. In this cross-sectional study, 26 patients on dialysis underwent a ^18^F-NaF PET and a bone biopsy. Bone turnover-based classification was assessed using Malluche’s historical reference values for normal bone turnover. In unified turnover-mineralization-volume (TMV)-based classification, the whole histopathological picture was evaluated and the range for normal turnover was set accordingly. Fluoride activity was measured in the lumbar spine (L1–L4) and at the anterior iliac crest. On the basis of turnover-based classification of ROD, 12% had high turnover and 61% had low turnover bone disease. On the basis of unified TMV-based classification of ROD, 42% had high turnover/hyperparathyroid bone disease and 23% had low turnover/adynamic bone disease. When using unified TMV-based classification of ROD, ^18^F-NaF PET had an AUC of 0.86 to discriminate hyperparathyroid bone disease from other types of ROD and an AUC of 0.87, for discriminating adynamic bone disease. There was a disproportion between turnover-based classification and unified TMV-based classification. More research is needed to establish normal range of bone turnover in patients with CKD and to establish the role of PET imaging in ROD.

**Supplementary Information:**

The online version contains supplementary material available at 10.1007/s00223-021-00874-9.

## Introduction

As chronic kidney disease (CKD) progresses, a majority of the patients have abnormalities in mineral homeostasis referred to as renal osteodystrophy (ROD) [1–4]. Abnormalities are seen in bone turnover, mineralization and volume. Hyperparathyroid bone disease is defined as high turnover, with elevated osteoblast and osteoclast activities, increased osteoid width, and peritrabecular fibrosis. Adynamic bone disease is defined as low turnover with reduced osteoblast and osteoclast activities.

Bone biopsy with the following histomorphometric analysis is the gold standard for evaluation of ROD [1, 5, 6]. Bone histomorphometric parameters can be divided into structural and remodeling [7, 8]. Structural parameters measure bone mass and structure. Remodeling parameters include both static and dynamic parameters. Static parameters include bone volume (BV/TV, %) osteoid volume (OV/BV, %), osteoid thickness (O.Th, µm), eroded surface (ES/BS, %), osteoblast (Ob.S/BS, %), and osteoclast surfaces (Oc.S/BS, %). Dynamic parameters yield information on bone formation rate (BFR/BS, mm^3^/cm^2^/year), activation frequency (Ac.f, 1/year), mineralizing surface (MS/BS, %), and mineralization lag time (Mlt, days). The measurement of dynamic parameters is possible only after labelling with tetracycline. Turnover is defined based on bone formation rate and/or activation frequency [7, 8].

A few studies have tried to assess normal values for bone histomorphometric parameters [9–19]. Usually the histomorphometric findings vary even among healthy individuals. Among others, race, age, and gender may cause variance to the histomorphometric results, making it difficult to set range for normal values. According to the reported studies, the values of normal BFR differ in different populations. In a British study of 84 healthy men and women [18] and in the studies of Recker and co-workers [9, 10, 15], the BFR and activation frequency were substantially lower than the normal range reported in the studies of Malluche [2, 16, 17]. There also seems to be intra-individual variability in the microarchitecture in different sites of the skeleton [20].

In CKD patients, recent research has focused on finding a biomarker, which correlates with turnover [21–23]. Plasma parathormone (PTH) measurement is commonly used to evaluate these patients, and extremely high or low PTH levels may predict the underlying bone disorder [24, 25]. However, the ability of PTH to correctly estimate turnover in bone is limited [22, 26]. Biomarkers such as carboxy-terminal collagen crosslinks (CTX), procollagen type 1 N-terminal propeptide (PINP), and tartrate-resistant acid phosphatase 5b (TRAP5) have been evaluated [21, 22], but no biomarker in clinical use has yet been proven superior or more suitable than PTH to estimate overall bone histopathology.

Several noninvasive imaging methods, such as high-resolution peripheral computed tomography and magnetic resonance imaging, have been studied in patients with chronic kidney disease and mineral and bone disorder, but these are static imaging methods. ^18^F-Sodium Fluoride positron emission tomography (^18^F-NaF PET) is a noninvasive dynamic imaging technique that allows assessment of regional bone turnover [27–29]. ^18^F-Fluoride is a bone-seeking tracer, which reflects remodeling of bone with a half-life of 110 min [30]. ^18^F-NaF is the preferred imaging technology when studying quantitative molecular imaging of bone [31]. We have previously shown a clear correlation between histomorphometric markers and fluoride activity in the ^18^F-NaF PET scan in dialysis patients [32].

The aim of this cross-sectional study was to compare only bone turnover -based classification of ROD, where Malluche’s reference values of normal turnover were used, and the unified classification system that includes parameters of turnover (T), mineralization (M) and volume (V) (unified TMV-based classification). In the unified TMV-based classification also static parameters were included in addition to the dynamic ones (BFR and Ac.f) when setting the diagnosis of the subtypes of ROD. Both classifications were compared to ^18^F-NaF PET analysis. The hypothesis was that bone turnover-based classification of ROD correlates with the unified TMV-based classification and that the fluoride activity in the ^18^F-NaF PET correlates with both classifications.

## Materials and Methods

The study was approved by the Ethics committee of the Hospital District of South Western Finland and was conducted in accordance with the Declaration of Helsinki as revised 1966. The study is registered in ClinicalTrials.gov protocol registration and result system. All subjects gave written informed consent.

### Study Subjects

Patients with end-stage renal disease were recruited from the Kidney center unit in Turku. The study group is the same as in the previous publication^32^, except for one more included patient and one excluded by the histomorphometrist (the bone biopsy did not reach required standards). All the bone biopsies were re-evaluated. The inclusion criteria were: dialysis vintage for at least 3 months and biochemical abnormalities; long-term elevated PTH and hyperphosphatemia, indicating mineral and bone disorder. Exclusion criteria were: pregnancy, previous parathyroidectomy, and bisphosphonate medication in the past 6 months. Ongoing medication for secondary hyperparathyroidism was continued. During the study period, the medication remained unchanged. All patients underwent a ^18^F-NaF PET scan, and a bone biopsy was performed within 4–6 weeks after the PET scan The bone biopsy was performed as a part of the study protocol. Biochemical markers were obtained right before dialysis sessions or on the morning of the bone biopsy.

In addition, seven healthy subjects were recruited as a validation group for the PET imaging. The healthy subjects underwent a ^18^F-NaF PET scan after assessment of routine laboratory tests to rule out underlying kidney or bone disease. No bone biopsy was performed.

The PET scans and bone biopsies were obtained during 2016–2019.

### Laboratory Assessment

Serum ionized calcium, alkaline phosphatase, phosphate, 25-Hydroxyvitamin D, 1,25-dihydroxyvitamin D, intact parathormone, albumin, acid–base balance, full blood count, and creatinine were performed in all patients. Coagulation screen was obtained previous to the bone biopsy. All tests were performed and analyzed by the local University Hospital laboratory.

### Bone Biopsy and Histomorphometry

Iliac crest biopsies were performed vertically under local anesthesia including one cortex. All patients underwent fluorochrome double labeling by receiving 500 mg tetracycline three times daily for 2 days per os, followed by a drug free interval of ten days and a further 2 days administration of tetracycline. Bone biopsy was completed 7–10 days after the second label. The investigator double-checked before the procedure that tetracycline was taken as ordinated. Bone biopsies were obtained using a Snarecoil Mermaid Medical RBN-86 8G (3.3 mm) × 15c m needle. All the patients underwent a successful bone biopsy procedure without complications.

Bone biopsies were fixed in 70% ethanol for at least 48 h before embedding in polymethylmethacrylate. The samples were cut into 5-μm thick sections and then stained with modified Masson–Goldner trichrome stain for static parameters, unstained sections were used for dynamic parameters. A semiautomatic image analyzer (BioquantOsteoII, Bioquant Image Analysis Corporation, Nashville, TN, USA) was used for analyzing all parameters.

In two patients, with only a single tetracycline label, we used a value for MAR of 0.3 µm/day in line with ASBMR Histomorphometry Nomenclature Committee recommendations for biopsies with only single labels [8].

In the bone turnover -based classification of renal osteodystrophy, we used Mallcuche´s reference values for normal turnover: bone turnover was classified as normal when Ac.f was between 0.49 and 0.72/year and/or BFR/BS was 18.0–38.0 µm/year [2, 13, 17].

In the unified TMV -based classification of renal osteodystrophy, the whole histopathological picture was evaluated, i.e. bone formation rate, activation frequency and mineralized surfaces as well as osteoblast- and osteoclast activities, osteoid width, eroded surfaces and the existence of peritrabecular fibrosis [33]. The values for normal turnover were set using the results of Recker et co (mean ± 1SD) [9, 10, 15]. The range for normal turnover in men was: BFR/BS 3.6–18.8 µm/year and Ac.f 0.12–0.6, in postmenopausal women: BFR/BS 6–22 µm/year and Ac.f 0.11–0.49/year and in premenopausal women: BFR/BS 3–13 µm/year and Ac.f 0.04–0.26/year.

All samples were analyzed by an independent histomorphometrist (HK). The histomorphometrist was blinded to the clinical history and details of the study subjects and to the PET-results.

### ^18^F-Fluoride Positron Emission Tomography

The PET scans were acquired using a Discovery VCT scanner (GE Healthcare). The tracer ^18^F-Fluoride ([^18^F]F^−^) is produced by 11-MeV proton irradiation of ^18^O-water using a cyclotron. The quality control tests for the ^18^F-NaF are conforming to the European Pharmacopeia. The subjects were positioned supine with the lumbar vertebrae in the field of view. A 60 min scan of the lumbar spine (L1–L4) followed by a 10 min static scan of the pelvis was done. The 60 min dynamic scan was begun simultaneously with an intravenous injection of 200 MBq ^18^F-NaF. The dynamic scan consisted of twenty-four 5-s, four 30-s and fourteen 240-s time frames. Low-dose CT-scans were done for image segmentation and attenuation correction. To generate bone activity curves (kilo becquerels per milliliter), regions of interest (ROI) in the lumbar spine were defined by drawing a ROI within each vertebral body, avoiding the end-plates and disk space. In the static PET scan of the pelvis ROI was defined by drawing a ROI on the anterior iliac crest, in the same region the bone biopsy was later obtained. Values were calculated both from the right and the left anterior iliac crest and a mean value was calculated. It is necessary to measure the arterial input function to calculate the plasma clearance of fluoride to bone. Also in this study we used an image derived input function by placing a ROI over the abdominal aorta (arterial input function, AIF) [34–36]. Image derived AIFs can present technical challenges and extra caution was attended when drawing the aorta ROI. The picture frames and the injected tracer´s activity were determined together with the physicist in charge of the VCT scanner, so that reconstruction produces quantitative image voxel values in all time frames. Patlak analysis was used to estimate the plasma clearance of ^18^F-Fluoride (net influx rate, K_i_) into the bone at the lumbar spine [37]. For the static scan of the pelvic bone; fractional uptake rate (FUR), which is an approximation of Patlak K_i_ [37], was calculated by dividing the bone activity concentration by area-under-curve of blood activity from ^18^F-Fluoride administration time to the time of static scan. Activity measurements were corrected for radioactive decay to the time of injection.

### Statistical Analysis

Statistical analyses for background variables were performed using SAS 9.4 for Windows and JMP Pro 14. Normality tests for bone histomorphometric and ^18^F-NaF PET were done visually together with the Shapiro–Wilk test. Many of the parameters failed the normality test and nonparametric statistical tests were used. Characteristics of the study population were expressed as median and interquartile range (IQR) or mean and standard deviation (SD). Correlations between bone turnover parameters and fluoride activity in the PET scan were assessed using the Spearman rank correlation test. For estimating the difference between means in different groups we used one-way analysis of variance (ANOVA) after logarithmic transformation and for pairwise comparison of different groups, we used Tukey's method. Histomorphometric parameters and fluoride activity in the PET scan were compared based on turnover using Wilcoxon test. Kappa statistics was calculated to estimate reliability of two methods. We assessed the receiver operating characteristics (ROC) curve for log transformed data. Based on the ROC curve we obtained the area under the curve (AUC) using trapezoidal rule and calculated sensitivity, specificity and positive and negative predictive values. Cut off values were calculated as optimal cut off values in this dataset. AUC of 0.6–0.7 was considered as poor, 0.7–0.8 as fair, 08–09 as good and 0.9–1 as excellent. A *p* value of 0.05 (two-tailed) or less was considered statistically significant.

## Results

### General

The characteristics of the study group is shown in Table 1. All 26 patients were of Caucasian race, the average age was 66 years. Median dialysis vintage was 10 months. Laboratory parameters and medication are shown in Table 1. Of 32 eligible patients, 5 were excluded because of insufficient bone biopsy and one because of problems with data transmission of the PET imaging. The flow diagram is shown in Figure S1 in the supplements. A clear correlation between fluoride activity in the PET scan and histomorphometric parameters (both dynamic and static) is shown in Table S1 in the supplements. There was also a clear correlation between fluoride activity and PTH, but not between fluoride activity and tALP, Table S1.

**Table 1:** Characteristics of the study group

No. of patients	26
Female sex (%)	13 (50)
Age, year (median, range)	66 (37–83)
BMI (mean, SD)	23.9 ± 3.5
Smoker (%)	6 (23)
History of diabetes (%)	9 (35)
Dialysis vintage, month (median, range)	10 (6–37)
Laboratory parameters	
fS-calcium-ion 1.16–1.13 mmol/l (median, IQR)	1.17 (1.11–1.23)
fP-phosphorus 0.71–1.23 mmol/l (median, IQR)	1.61 (1.41–1.99)
fP-PTH 15–65 ng/l (median, IQR)	285 (178–537)
P-D-25 > 50 nmol/l (median, IQR)	70 (40–94)
S-D-125 37–216 pmol/l (median, IQR)	30 (24–58)
P-tALP 35–105 U/l (median, IQR)	88 (67–132)
P-Alb 36–45 g/l (median, IQR)	31.9 (27.8–33.6)
Medication	
Calcimimetic (%)	4 (15)
Alfacalcidol, Paricalcitol (%)	14 (58)
Calcium carbonate (%)	22 (85)
Cholecalciferol (%)	23 (88)
Sevelamer/lantane carbonate (%)	16 (62)
Corticosteroid (%)	2 (8)

The mean age in the control group was 68 year (range 42–77 year). 57% were females.

### Histomorphometric Results: Turnover-based vs Unified TMV-based Classification of ROD

**Fig. 1:**
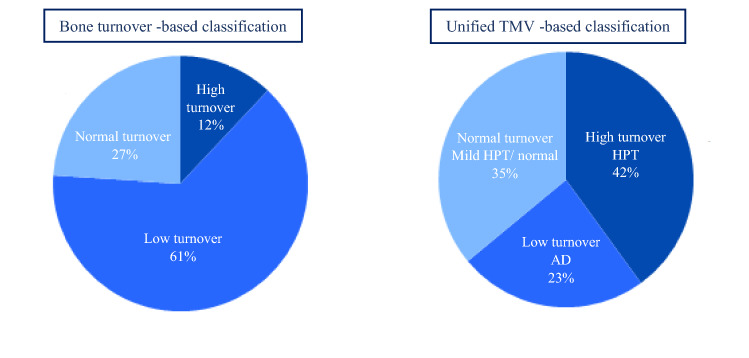
Distribution of renal osteodystrophy categories—bone turnover-based versus unified TMV-based classification

On the basis of turnover-based classification of ROD, when using Malluche’s definition of turnover, 12% of the study population had high turnover and 61% low turnover. On the basis of unified TMV-based classification of ROD, 42% had hyperparathyroid bone disease/high turnover and 23% adynamic bone disease/low turnover (Fig. 1). Two patients with low turnover also had a clear mineralization defect based on the bone biopsy. In the group of normal/mild hyperparathyroid bone disease with normal turnover, two patients had overall normal findings and two patients had a mineralization defect.

In Table 2a and b are shown bone histomorphometric parameters according to turnover-based and unified TMV -based classification of ROD. In the supplements Table S1a and S1b is shown pairwise comparison of the groups in both types of classifications. The unified TMV-based classification of ROD is statistically associated with turnover-based classification (*p* < 0.001). In cases of low turnover/adynamic bone disease, all cases match, but in case of hyperparathyroid bone disease only 3 cases of 11 matched. Patients with normal turnover/normal bone or mild hyperparathyroid bone disease were classified as low turnover when using Malluche’s reference values, and patients with high turnover/hyperparathyroid bone disease were classified as normal turnover, Table 3.

**Table 2:** Histomorphometric and imaging parameters in dialysis patients according to distribution of renal osteodystrophy categories–turnover-based (a) and unified TMV-based (b) classification

a—Turnover-based classification	High turnover (*n* = 3)	Normal turnover (*n* = 7)	Low turnover (*n* = 16)	*p* value
BFR/BS (µm^3^/µm^2^/year)	35.0 (33.9–39.3)	24.8 (20.2–30.0)	7.5 (53–12.5)	< 0.001
Ac.f (1/year)	0.82 (0.67–0.83)	0.49 (0.46–0.57)	0.19 (0.15–0.31)	< 0.001
Oc.S/BS (%)	3.5 (1.4–6.7)	2.5 (1.4–3.3)	0.8 (0.11–1.7)	0.02
Ob.s/BS (%)	7.2 (3.2–16.9)	4.9 (3.2–14.0)	2.0 (0.2–4.6)	0.005
Mlt (d)	31.4 (22.3–34.8)	33.8 (25.5–35.9)	57.6 (33.1–100.5)	0.05
MS/BS (%)	9.5 (9.4–10.7)	6.5 (5.1–9.2)	2.9 (2.0–5.5)	0.002
O.th (µm)	8.7 (7.2–10.0)	7.4 (6.1–10.6)	5.7 (5.0–6.8)	0.02
MAR (µm/day)	1.01 (0.99–1.01)	1.03 (0.8–1.2)	0.7 (0.6–0.9)	0.008
OS/BS (%)	38.4 (24.1–40.8)	27.5 (20.9–36.6)	24.8 (19.3–31.8)	0.27
ES/BS (%)	4.0 (2.4–6.9)	3.8 (2.8–4.8)	1.6 (0.7–2.9)	0.03
OV/BV (%)	6.8 (5.5–7.8)	3.5 (3.4–5.7)	3.0 (2.4–4.0)	0.02
BV/TV (%)	18.2 (18.1–25.0)	22.8(18.8–27.2)	18.7 (14.1–24.9)	0.56
Mean K_i_ (L1-L4) mL/min/mL	0.067 (0.055–0.077)	0.053 (0.032–0.059)	0.038 (0.031–0.045)	0.02
Mean FUR (hip) mL/min/mL	0.065 (0.050–0.066)	0.056 (0.041–0.073)	0.039 (0.032–0.046)	0.01

b—Unified TMV-based classification	High turnover—HPT (*n* = 11)	Normal turnover—mild HPT/normal (*n* = 9)	Low turnover AD (*n* = 6)	*p* value
BFR/BS (µm^3^/µm^2^/year)	26.0 (20.2–34.0)	9.7 (7.5–16.3)	5.2 (2.3–5.7)	< 0.001
Ac.f (1/year)	0.56 (0.46–0.67)	0.25 (0.16–0.39)	0.13 (0.11–0.17)	< 0.001
Oc.S/BS (%)	2.5 (1.4–3.5)	0.9 (0.23–1.31)	0.7 (0.001–1.9)	0.01
Ob.s/BS (%)	4.9 (3.2–14.0)	2.9 (2.0–4.0)	0.12 (0.001–0.3)	< 0.001
Mlt (d)	33.8 (25.5–35.9)	44.4 (30.3–79.6)	106.7 (99.6.0–174)	0.009
MS/BS (%)	6.6 (5.1–9.5)	3.2 (2.6–6.9)	1.9 (1.4–2.7)	< 0.001
O.th (µm)	7.4 (6.6–10.0)	5.6 (4.5–6.7)	5.5 (4.9–6.7)	0.02
MAR (µm/day)	1.01 (0.83–1.1)	0.7 (0.6–0.9)	0.7 (0.3–0.8)	0.007
OS/BS (%)	27.5 (22.0–37.5)	23.2 (17.3–32.3)	27.4 (19.4–34.7)	0.60
ES/BS (%)	3.8 (2.8–4.8)	1.9 (0.90–3.2)	1.5 (0.09–2.1)	0.01
OV/BV (%)	5.1 (3.4–6.1)	3.6 (2.1–4.6)	2.6 (2.2–5.2)	0.09
BV/TV (%)	22.8 (18.2–27.2)	17.7 (12.7–23.1)	18.9 (14.7–25.8)	0.38
**Mean K**_**i**_** (L1-L4)** mL/min/mL	0.056 (0.051–0.067)	0.039 (0.037–0.047)	0.032 (0.026–0.037)	0.003
**Mean FUR (hip)** mL/min/mL	0.060 (0.050–0.071)	0.041 (0.035–0.049)	0.032 (0.029–0.038)	0.002

Data are presented as median (interquartile range)

In the turnover-based classification, Malluche´s reference values for normal turnover were used: BFR/BS 18–38 µm/y and Ac.f 0.49–0.74/year

In the unified TMV-based classification, reference values for normal turnover (Recker et co, mean ± 1SD) in men was: BFR/BS 3.6–18.8 µm/year and Ac.f 0.12–0.6, in postmenopausal women: BFR/BS 6–22 um/year and Ac.f 0.11–0.49/year and in premenopausal women: BFR/BS 3–13 µm/y and Ac.f 0.04–0.26/year

Mean K_i_ (L1-L4) reflects the fluoride activity in the PET scan in the lumbar spine and Mean FUR (hip) the fluoride activity at the anterior iliac crest. *p* < 0.05 is statistically significant

*BFR/BS* bone formation rate per bone surface, *Oc.S/BS* osteoclast surface per bone surface, *Ob.S/BS* osteoblast surface per bone surface, *MAR* mineral apposition rate, *Mlt* mineralization lag time, *MS/BS* mineralized surface per bone surface, *O.th* osteoid thickness, *Ac.f* activation frequency per year, *OS/BS* osteoid surface per bone surface, ES/BS erosion surface per bone surface, *OV/BV* osteoid volume of bone volume, *BV/TV* bone volume of tissue volume

**Table 3:** Association between turnover-based and unified TMV-based classification of ROD

Turnover-based classification	Unified TMV-based classification			
	Low turnover—AD	Normal turnover—mild HPT/normal	High turnover—HPT	Total
Low turnover	6	9	1	16
Normal turnover	0	0	7	7
High turnover	0	0	3	3
Total	6	9	11	26

In cases of low turnover/adynamic bone disease all the subjects matched to turnover-based classification. In cases of hyperparathyroid bone disease, only 27% of the subjects matched

Of 26 subjects, only nine matched, 34% (Kappa’s test was 0.19). Patients with normal turnover/normal bone or mild hyperparathyroid bone disease were classified as low turnover and patients with high turnover/hyperparathyroid bone disease were classified as normal turnover when using Malluche’s reference values for normal bone turnover

*AD* adynamic bone disease, *HPT* hyperparathyroid bone disease

### PET-Studies

**Fig. 2:**
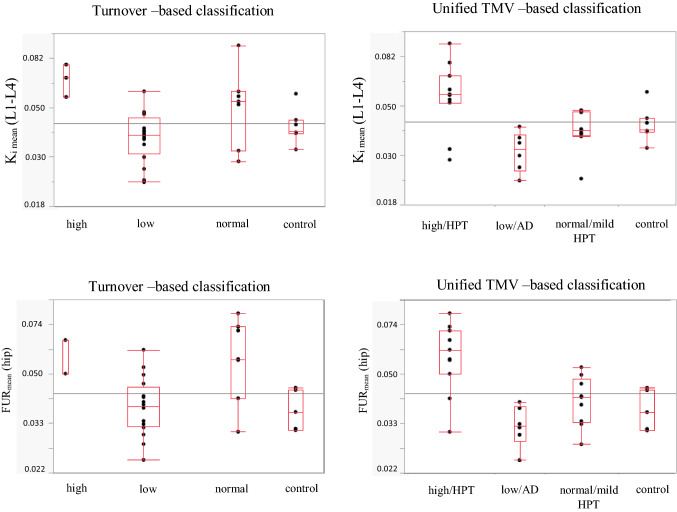
Fluoride activity in the lumbar spine and at the anterior iliac crest in the control group and according to turnover-based and unified TMV-based classification of ROD. Tukey’s box-plot figure illustrates the fluoride activity in the control group and in the two classification groups. *K*_*i mean*_* (L1–L4)* fluoride activity in the lumbar spine, *FUR*_*mean*_* (hip)* fluoride activity at the anterior iliac crest, *HPT* hyperparathyroid bone disease

In Fig. 2 is shown the fluoride activity in the lumbar spine and anterior iliac crest in the different categories of ROD, both according to turnover-based and TMV-based classification, and the fluoride activity in the control group. The healthy subjects fluoride activity in the lumbar region (K_i mean_) was 0.039 (0.038–0.044) mL/min/mL and at the anterior iliac crest (FUR_mean_) 0.037 (0.032–0.044) mL/min/mL ^32^, which correlates well with the fluoride activity for normal turnover in the unified TMV-classification: 0.039 (0.037–0.047) in the lumbar spine (K_i mean_) and 0.041 (0.035–0.049) at the anterior crest (FUR_mean_). In turnover-based classification fluoride activity in the lumbar region (k_i mean_) for normal turnover was 0.053 (0.032–0.059) and at the anterior iliac crest 0.056 (0.041–0.073).

### Diagnostic Accuracy of 18F-NaF PET for High Turnover/Hyperparathyroid Bone Disease

In the ^18^F-NaF PET scan, hyperparathyroid bone disease was defined as fluoride activity higher than the cut-off value (0.055 mL/min/mL) in the lumbar region or at the anterior iliac crest. To test ^18^F-NaF PET imaging as a diagnostic tool, we defined the ROC-curve. In ROC analysis for discriminating high turnover/hyperparathyroid bone disease from other types of ROD, using unified TMV-based classification, fluoride activity in the PET scan had an AUC of 0.86, the sensitivity was 82% and specificity 100%, the negative predictive value 88% and positive predictive value 100%, Table 4a.

**Table 4:** 18F-NaF PET’s diagnostic accuracy in ROD

Variables	AUC		Criterion	Sensitivity (%)	Specificity (%)	NPV (%)	PPV (%)
a. 18F-NaF PET strength to recognize high turnover/hyperparathyroid bone disease							
^18^F-fluoride activity in the PET scan unified TMV-based	0.86		Cut-off > 0.055 Ml/min/Ml	82	100	88	100
PTH unified TMV-based	0.69		> 450 ng/ml	55	87	72	75
b. 18F-NaF PET strength to recognize low turnover/adynamic bone disease							
^18^F-fluoride activity in the PET scan unified TMV-based		0.87	Cut-off < 0.038 Ml/min/Ml	100	70	100	50
^18^F-fluoride activity in the PET scan turnover -based		0.83	Cut-off < 0.038 Ml/min/Ml	63	80	57	83
PTH—unified TMV-based		0.78	< 185 ng/ml	67	85	89	57
PTH—turnover-based		0.68	< 185 ng/ml	31	80	42	71

When classification of ROD was done based on Malluche’s reference values of normal turnover, the ROC curve could not be defined for patients with high turnover, because of the scarcity of patients.

When assessing ROC analysis for PTH to discriminate high turnover/hyperparathyroid bone disease from other types of ROD, PTH had an AUC of 0.69, cut-off for PTH was set at 450 ng/ml. Sensitivity was 55% and specificity 87%, Table 4a.

### Diagnostic Accuracy of ^18^F-NaF PET for Low Turnover/Adynamic Bone Disease

In the ^18^F-NaF PET scan, adynamic bone disease was defined as fluoride activity below the cut-off value (0.038 mL/min/mL) in the lumbar region or at the anterior iliac crest. The ROC was defined as explained above. In ROC analysis for discriminating low turnover/adynamic bone disease from other types of ROD, using unified TMV-based classification, fluoride activity in the PET scan had an AUC of 0.87 with 100% sensitivity and 70% specificity, the negative predictive value was 100% and positive predictive value 50% Table 4b.

When classification of ROD was done based on turnover –based classification (cut-off value 0.038 mL/min/mL), the sensitivity of the PET imaging to differentiate between low turnover and non-low turnover was 63%, and specificity 80%, AUC was 0.83. Negative predictive values was 57% and positive predictive value 83%

When assessing ROC analysis for PTH for discriminating low turnover/adynamic bone disease, PTH had an AUC of 0.78. When cut-off for PTH was set at 180 ng/ml, sensitivity was 67% and specificity 85%, Table 4b. When using turnover-based classification of ROD to discriminate between low turnover and non-low turnover PTH had an AUC of 0.68, sensitivity was 31% and specificity 80%, Table 4b.

## Discussion

This study shows a clear disproportion between turnover-based classification and unified TMV -based classification of ROD. We are, to our knowledge, the first to report the difference, when classifying the subtypes of ROD in these two ways and to compare the results to PET imaging. In this study population, 61% of the patients had low turnover based on Malluche’s range of normal bone turnover, and only 12% high turnover. Based on unified TMV -based classification, in which the whole histopathological picture, and the results of the studies of Recker [9, 10, 15] was taken into account when defining normal turnover, 23% had low turnover/adynamic bone disease and 42% had high turnover/hyperparathyroid bone disease. PTH´s diagnostic accuracy improved, when using unified TMV-based classification of ROD as reference.

Malluche has stated that every laboratory should define the range of normal values of quantitative histomorphometric parameters of their own [17]. It is understandable that this is not easily accomplished. The historical reference values of Malluche for normal turnover (dynamic parameters) are based on bone biopsies taken in the 1980s from 14 healthy men and 14 healthy women, ages 20–83 years [16]. In the studies of Recker and co-workers published in 2018, altogether 96 healthy men and women underwent bone biopsies [9, 15]. They found the range of normal values of turnover to be substantially lower than in Malluche´s definition of normal turnover. Many histomorphometric values show differences in men and women [9, 10, 15, 18]. The results of dynamic and static parameters, such as osteoblast- and osteoclast activities also show variation with age. Remodeling increases in women after menopause, with increase in osteoclast activity and decrease in bone formation rate and osteoblast activity [15, 18, 19]. Therefore, the use of age and sex adjusted values of turnover might be more feasible.

The fluoride activity in the ^18^F-NaF PET scan in the control group matched the fluoride activity in the group with normal turnover/mild hyperparathyroid bone disease, when the classification of ROD was done based on the unified TMV-based classification. This indicates, that in this study population, the use of Malluche’s reference values of normal turnover overestimates the number of low turnover and underestimates the number of high turnover. It is also noteworthy, that in clinical practice, the unified TMV-based classification of the bone biopsy and the statement of the histomorphometrist, guide medical decision, not only BFR or/and Ac.f.

Fluoride activity in the ^18^F-NaF PET scan correlates well with dynamic histomorphometric markers in the bone biopsy and with several static markers as well [32]. In our recent publication [32], we used Malluche’s reference values, being the most cited in this field of nephrological research. When using the unified TMV-based classification, the cut-off for tracer activity was set at 0.038 mL/min/mL, which also matches the median fluoride activity for the control group. It is noteworthy that with the unified TMV –based classification, the diagnostic accuracy of ^18^F-NaF PET to differentiate the subtypes of ROD, improves. The tracer ^18^F-Fluoride reflects osteoblast activity and bone remodeling, i.e. the metabolic activity in the bone. ^18^F-NaF PET specificity to recognize high turnover/hyperparathyroid bone disease is 100% and sensitivity 82%, with no false positive cases. ^18^F-NaF PET sensitivity to recognize low turnover/ adynamic bone disease is 100%, with no false negative cases. Moreover, the specificity and sensitivity of ^18^F-NaF PET was superior to PTH to diagnose high turnover/ hyperparathyroid bone disease. ^18^F-NaF PET sensitivity to diagnose low turnover/adynamic bone disease was also superior to PTH.

These results suggest that PET imaging could work as a diagnostic tool to confirm high turnover/hyperparathyroid bone disease before parathyroidectomy or rule out low turnover/adynamic bone disease before initiating antiresorptive medication in case of low energetic fracture. However, more research is needed before these results can be adapted in clinical practice. It is important to understand that ^18^F-NaF PET measures turnover and bone remodeling and cannot, at least based on the knowledge we currently have, discriminate between patients with a mineralizing defect.

Recent research has focused on finding a biomarker that reflects bone turnover also in CKD patients. One challenge has been to find a biomarker that correlates with turnover and is superior to PTH, which is the main biomarker used for evaluating bone metabolism in CKD patients [2, 21, 22]. PTH´s sensitivity and specificity to estimate underlying bone turnover compared to histomorphometric findings in the bone biopsy is limited [22, 26]. In general, it is not unambiguous how well biomarkers, which reflect the overall bone formation in the skeleton, will correlate with bone histomorphometry from only one small site of the skeleton. Inconsistency between histomorphometric markers and bone mineral density after treatment with bisphosphonates has been observed in several osteoporosis studies [38, 39]. Several PET studies have shown regional differences in bone metabolism in different sites of the skeleton [40, 41], which support the assumption that bone metabolism varies at different sites. This emphasizes the challenge, when global markers of bone remodeling in CKD patients are developed and highlights the fact that PET imaging, which gives a more extensive picture of the skeleton, could be a feasible method.

The limitation of this study is the small sample size, and the use of only PTH as biomarker in analyses. The bone biopsies were taken vertically from the anterior iliac crest. This is a less invasive procedure for the patient. Vertical biopsy is technically easy for the physician but could possibly have an impact on the interpretability and comparability of studies in which the bone biopsies were taken transiliacly. However, the distribution of unified TMV-based subtypes of ROD was the same as in a previous publications of bone histomorphometry in the Finnish population [42]. One limitation is also, that the control group did not undergo a bone biopsy and was not specifically matched to the study population.

The results of this study raise several important questions. What reference values of normal bone turnover should be recommended to use in the CKD population? Should the reference values be gender and age adjusted? A discussion is needed to define reference values for normal turnover in CKD patients.

In conclusion we showed a clear disproportion between only turnover–based and unified TMV-based classification of ROD. ^18^F-NaF PET’s ability to differentiate between the subtypes of ROD improved, when the whole histopathological picture was evaluated. More research is needed to define the normal range of bone turnover in different populations and to establish the role of PET imaging in diagnostic settling of ROD.

## Supplementary Information

Below is the link to the electronic supplementary material.

Supplementary file1 (DOCX 79 kb)

## Data Availability

The data that support the findings of this study are available from the corresponding author upon reasonable request.
