# Supplementary material for: Bone Histomorphometry and 18F-Sodium Fluoride Positron Emission Tomography Imaging: Comparison Between only Bone Turnover-based and Unified TMV-based Classification of Renal Osteodystrophy
Source: Calcif Tissue Int. 2021 Jun 17;109(6):605–14. doi: 10.1007/s00223-021-00874-9 (PMC8531121; doi:10.1007/s00223-021-00874-9)

**Bone Histomorphometry and ^18^F- Sodium Fluoride Positron Emission Tomography Imaging - Comparison between only bone turnover -based and unified TMV -based classification of renal osteodystrophy**

Louise Aaltonen, MD^1^, Niina Koivuviita, M.D, Ph.D^1^, Marko Seppänen, M.D., Ph.D^2,3^, Inari S Burton, M.D., Ph.D^4^, Heikki Kröger, M.D, Ph.D^4,5^, Eliisa Löyttyniemi, M.D, Ph.D^6^ and Kaj Metsärinne, M.D, Ph.D^1^.

^1^Kidney Center, Department of Medicine, Turku University Hospital, PL 52, Kiinamyllynkatu 4-8, Turku 20521, Finland, ^2^Turku PET Centre, University of Turku, Kiinamyllynkatu 4-8, Turku 20521, Finland, ^3^ Department of Clinical Physiology, Nuclear Medicine, Turku University Hospital, PL 52, Kiinamyllynkatu 4-8, Turku 20521, Finland, ^4^Kuopio Musculoskeletal Research Unit (KMRU) Institute of Clinical Medicine, University of Eastern Finland, POB 1627 Kuopio Finland, ^5^Kuopio University Hospital, Kuopio, Finland, ^6^Department of Biostatistics, University of Turku, Kiinamyllynkatu 10, 20014 Turku, Finland

**Running title:** Bone Histomorphometry and ^18^F-NaF-PET

**Correspondence:** Louise Aaltonen, Kiinamyllynkatu 4-8, 20521 Turku, +358504003175, louise.aaltonen@tyks.fi

**Table S1**. Correlation between fluoride activity in the ^18^F-NaF PET scan and histomorphometric and laboratory parameters.

|  | **K_i mean_ (L1 – L4)** | **FUR _mean_ (hip)** |
| --- | --- | --- |
| BFR/BS (µm/year) | r_s_ = 0.63 p <0.001 | r_s_ = 0.66 p <0.001 |
| Ac.F (1/year) | r_s_ = 0.63 p = 0.001 | r_s_ = 0.64 p <0.001 |
| Oc.S/BS (%) | r_s_ = 0.66 p <0.001 | r_s_ = 0.65 p <0.001 |
| Ob.s/BS (%) | r_s_ = 0.52 p = 0.007 | r_s_ = 0.60 p = 0.001 |
| MS/BS (%) | r_s_ = 0.66 p = 0.008 | r_s_ = 0.50 p <0.009 |
| MAR (µm/day) | r_s_ = 0.44 p = 0.02 | r_s_ = 0.53 p <0.006 |
| OS/BS (%) | r_s_ = 0.66 p <0.001 | r_s_ = 0.66 p <0.001 |
| ES/BS (%) | r_s_ = 0.61 p < 0.001 | r_s_ = 0.60 p = 0.001 |
| Mlt (d) | p = 0.25 | p = 0.32 |
| O.Th (µm) | p = 0.12 | r_s_ = 0.46 p = 0.02 |
| tALP | p = 0.36 | p = 0.42 |
| PTH | r_s_ = 0.63 p <0.001 | r_s_ = 0.66 p <0.001 |

Table S1. K_i_ _mean_ (L1-L4) reflects the fluoride activity in the PET scan in the lumbar spine and FUR_mean_ (hip) the fluoride activity at the anterior iliac crest.

BFR/BS = bone formation rate per bone surface, Ac.f = activation frequency per year, Oc.S/BS = osteoclast surface per bone surface, Ob.S/BS = osteoblast surface per bone surface, MS/BS = mineralized surface per bone surface,  MAR = mineral apposition rate, OS/BS = osteoid surface per bone surface, ES/BS = erosion surface per bone surface, Mlt = mineralization lag time, O.th = osteoid thickness, OV/BV = osteoid volume of bone volume, BV/TV = bone volume of tissue volume. tALP = total alkaline phosphatase and PTH = parathyroid hormone. p < 0.05 is statistically significant.

**Table S1**. Correlation between fluoride activity in the ^18^F-NaF PET scan and histomorphometric and laboratory parameters.

Table S1. K_i_ _mean_ (L1-L4) reflects the fluoride activity in the PET scan in the lumbar spine and FUR_mean_ (hip) the fluoride activity at the anterior iliac crest.

BFR/BS = bone formation rate per bone surface, Ac.f = activation frequency per year, Oc.S/BS = osteoclast surface per bone surface, Ob.S/BS = osteoblast surface per bone surface, MS/BS = mineralized surface per bone surface,  MAR = mineral apposition rate, OS/BS = osteoid surface per bone surface, ES/BS = erosion surface per bone surface, Mlt = mineralization lag time, O.th = osteoid thickness, OV/BV = osteoid volume of bone volume, BV/TV = bone volume of tissue volume. tALP = total alkaline phosphatase and PTH = parathyroid hormone. p < 0.05 is statistically significant.

**Table S2**. p-value pairwise for comparison of differents groups - bone turnover -based (a) and unified TMV -based (b) classification of renal osteodystrophy.

**Table S2**. p-value pairwise for comparison of differents groups - bone turnover -based (a) and unified TMV -based (b) classification of renal osteodystrophy.

| **Table S2a**  **Turnover -based** | **High turnover vs**  **Normal turnover** | **High turnover vs Low turnover** | **Normal turnover vs Low turnover** |
| --- | --- | --- | --- |
| BFR/BS (µm^3^/µm^2^/1 day) | **0.01** | **0.001** | **0.001** |
| MAR (µm/day) | 0.97 | 0.05 | **0.02** |
| Oc.S/BS (%) | 0.24 | **0.004** | 0.05 |
| Ob.s/BS (%) | 0.75 | **0.02** | **0.02** |
| Mlt (d) | 0.99 | 0.18 | 0.07 |
| MS/BS (%) | 0.34 | **0.001** | 0.004 |
| O.Th (µm) | 0.76 | **0.03** | 0.03 |
| Ac.F (1/year) | **0.003** | **<0.001** | **<0.001** |
| OS/BS (%) | 0.61 | 0.43 | 0.95 |
| ES/BS (%) | 0.99 | 0.32 | 0.10 |

| **Table S2b**  **Unified TMV -based** | **HPT vs**  **Norm/mild HPT** | **HPT vs**  **AD** | **Norm/mild HPT vs AD** |
| --- | --- | --- | --- |
| BFR/BS (µm^3^/µm^2^/1 day) | **0.002** | **<0.001** | **0.002** |
| MAR (µm/day) | **0.1** | **0.004** | 0.23 |
| Oc.S/BS (%) | 0.39 | 0.26 | 0.90 |
| Ob.s/BS (%) | 0.71 | **<0.001** | **<0.001** |
| Mlt (d) | 0.12 | **<0.001** | **0.002** |
| MS/BS (%) | **0.03** | **<0.001** | **0.008** |
| O.Th (µm) | **0.01** | 0.05 | 0.95 |
| Ac.F (1/year) | **<0.001** | **<0.001** | **0.02** |
| OS/BS (%) | 0.82 | 0.96 | 0.96 |
| ES/BS (%) | 0.31 | **0.03** | 0.38 |

Table S2. AD = adynamic bone disease, HPT = hyperparathyreoid bone disease.

Table S2. AD = adynamic bone disease, HPT = hyperparathyreoid bone disease.


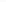

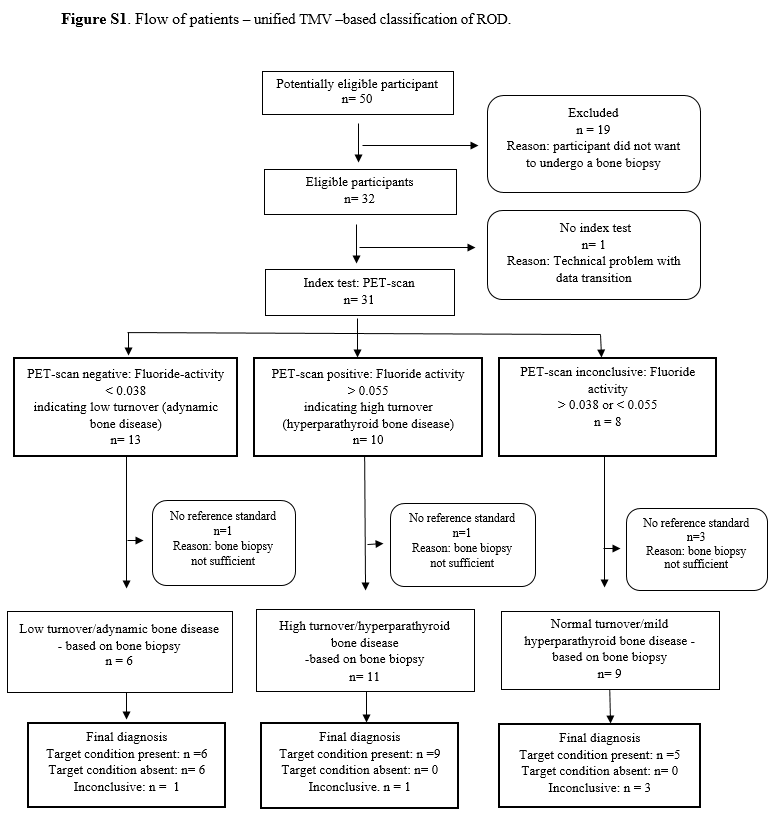

Supplement: Supplementary file 1 — Supplementary file1 (DOCX 79 kb) [file 223_2021_874_MOESM1_ESM.docx]
